# Vaccination: Its Natural History and Pathology

**Published:** 1900-03

**Authors:** 


**Vaccination: its Natural History and Pathology.** By S. Monckton Copeman, M.D, Pp. x., 257. London: Macmillan and Co., Limited. 1899.

Dr. Copeman's lectures of 1898 are of considerable interest and importance in relation to the ever-prominent question of compulsory vaccination. The earlier chapters are concerned with the history of the introduction of vaccination by Jenner, and a consideration of the sources of lymph. The histology of the vaccine vesicle and its bacteriology are then considered, and the history of animal vaccination and its introduction discussed. The remaining chapters are devoted to a careful consideration of the bacteriological purification and preservation of vaccine lymph by glycerine, with special investigations as to the action of glycerine on pathogenic and non-pathogenic micro-organisms. These chapters are fully illustrated by reproductions of photographs.

